# Will Boys Always Be Boys? The Criminalization of Street Harassment in Portugal

**DOI:** 10.1177/10778012221150276

**Published:** 2023-01-19

**Authors:** Beatriz Ribeiro

**Affiliations:** 1 112053 Faculty of Social Sciences and Humanities, Nova University of Lisbon, Lisbon, Portugal

**Keywords:** street harassment, gender violence, public policy, street-level bureaucrats, police

## Abstract

Albeit one of the most pervasive forms of gender violence, street harassment tends to be either not considered a crime or to be faulty criminalized. This investigation contributes to better understand the overall inefficiency of existing laws through an analysis of the criminalization of street harassment in Portugal. Particularly, it searches for obstacles to implementation among those responsible for the process—the street-level bureaucrats of the Portuguese Public Security Police. Through 14 semi-structured interviews, three groups of obstacles to implementation were identified: perceptions of the legislation's content, a masculinist institutional culture, and personal characteristics. These are new findings that contribute to an understanding of the perpetuation of gender violence through state's institutions and workers.

## Introduction

Albeit one of the most pervasive forms of gender violence (EIGE, n.d.; European Commission, n.d.; UN Women, 2018; WHO, 2012), an analysis of street harassment has been neglected when compared to other crimes like rape and domestic abuse.

Street harassment has multiple consequences for women. Besides causing physiological and/or physical harm, it can also lead to self-objectification (Fairchild & Rudman, 2008; Moya-Garófano et al., 2018), as the constant exposure of women to objectifying situations can lead to their internalization through body surveillance and self-inflicted body shaming (Fredrickson & Roberts, 1997). Furthermore, it creates gender differences in public spaces' usage: it hinders women's security in the public sphere and increases fear when using them (Macmillan et al., 2000; Mellgren and Invert, 2017). This results in a restriction of women's geographical mobility (Bowman, 1993; Fairchild & Rudman, 2008) and in their spatial exclusion (Junger, 1987; Koskela, 1999).

Yet, street harassment tends to be either not considered a crime^1^ or to be faulty criminalized (Fileborn & Vera-Gray, 2017; Kearl, 2015; Mellgren & Invert, 2017). Existing laws around the world seem inefficient; there appears to be no significant change in the number of street harassment's occurrences after its formal criminalization (Kearl, 2015). Some authors suggest this is due to a lack of victim's formal complaints (Fileborn & Vera-Gray, 2017; Lahsaeizadeh & Yousefinejad, 2011; Mellgren & Invert, 2017), a well-documented phenomenon in all gender-based violence's crimes (Felson & Paré, 2005; Johnson, 2012).

But instead of focusing on the victim's actions, this research proposes an analysis of the state's institutions and state's workers. By focusing on those responsible for implementation new answers to street harassment's flawed criminalization can emerge. Consequently, this investigation presents a case study of Portugal, where street harassment is considered a crime under Article 170° of the Penal Code. Particularly, it assesses the existence, or lack thereof, of obstacles to implementation among the police officers of the Portuguese Public Security Police—Polícia de Segurança Pública (PSP). These officers are the main state's employees responsible for the daily implementation of this Article, whose work involves direct interaction with citizens. Therefore, they fulfill Lipsky's (1980) definition of street-level bureaucrats, whose public policy theory provides the necessary theoretical framework to analyze PSP workers’ role in implementation, for it argues they have enough agency to influence the outcomes produced. A brief context on this theory, on street harassment in general and on its occurence in Portugal are what follows. Then, this study's methodology is presented, immediately followed by a display of the findings and subsequent discussion. Finally, this study ends with a conclusion summarizing the main findings and future investigation paths.

## Theoretical Framework

### Street Harassment

Disproportionately perpetrated by men against women, street harassment represents a form of violence against women (Lahsaeizadeh & Yousefinejad, 2011; Macmillan et al., 2000; Madan & Nalla, 2016). It is a worldwide reality affecting women from all continents. It can be defined as the set of verbal, nonverbal, and physical behaviors of a sexual nature, perpetrated in public sights like streets, sidewalks, roads, parks, and public transportation by someone unacquainted with the victim (Bowman, 1993). Some examples are saying “girl, baby, sweetheart,” whistling, making sexual comments, stalking, making unwanted sexual gestures or looks, masturbating in front of women, and touching/groping/spanking/pinching (Madan & Nalla, 2016), but, as Vera-Gray (2017) recently argued, it embodies all kinds of “male intrusions.”

Because it falls within a continuum of violence (Vera-Gray, 2017), street harassment contributes to the “shadow of sexual assault”^2^ (Fairchild & Rudman, 2008). This means women are concerned about being a victim of street harassment independently of their own experience with it (Mellgren & Ivert, 2019). Furthermore, this shadow also influences how women deal with the occurrence. The literature demonstrates how women prefer passive strategies instead of active strategies due to a fear of escalation (Dhillon & Bakaya, 2014; Olney, 2015). Thus, it is not only being the victim of street harassment that influences women's perception of security, but also the anticipation of its future occurrence and possible escalations.

Despite such consequences, not many scholars have turned to street harassment's widespread faulty criminalization. Thus far, literature highlighted the lack of victim's complaints to the police (Fileborn & Vera-Gray, 2017; Mellgren & Invert, 2017), a generalized phenomenon of all violence against women's crimes, particularly those of a sexual nature (Felson & Paré, 2005; Johnson, 2012). Indeed, rape literature defines this as one of its main attrition points (Lovett & Kelly, 2009). The same seems to happen with street harassment: for example, of the 1,941 women interviewed by Mellgren and Invert (2017), 98.5% did not report any prior experience with street harassment.

Some authors have suggested this stems from police forces not being particularly sensitive to gender crimes due a prevalence of gender-stereotyped attitudes and beliefs in the institution (Brown & King, 1998; Kearl, 2015; Meier & Nicholson-Crotty, 2006). Others, that the existence of an almost constant gender difference between the victims (the vast majority, women) and the officers (the vast majority, men) undermines the process of filling a complaint (Brown & King, 1998; Jamel, 2010; Kearl, 2015; Lockwood & Prohaska, 2015). Mellgren and Invert (2017) and Nielsen (2000) also showed how a large number of women do not come forward to file street harassment complaints because they do not believe the police would enforce existing laws. If the literature on rape indeed suggests gender-stereotyped attitudes and beliefs toward rape affect police officers' implementation of rape laws, the same could be happening with street harassment laws.

### Street Harassment in Portugal

Portugal verifies high levels of street harassment attrition—that is, there is a big gap between occurred cases of street harassment and people condemned it. As with any gender-based violence crime, street harassment's actual prevalence is difficult to establish and the number of cases reported in surveys/interviews remains the most reliable approximation (Felson & Paré, 2005; Johnson, 2012). The last national representative survey on violence against women in Portugal (Lisboa, 2009) concluded street harassment is the most prevalent form of sexual violence against women in the country, with an incidence of 38.4% (Patrício, 2009). Furthermore, Table 1 displays the total of street harassment's complaints received by the Associação Portuguesa de Apoio à Vítima (APAV)^3^ for the years 2016–2020.^4^ Although not the real number of street harassment's annual occurrences, it is clear it has a substantial presence in the country.

**Table 1.** Street Harassment's Complaints Received by the Associação Portuguesa de Apoio à Vítima (APAV) 2016–2020.

Year	Complaints (*N*)	Complaints (%)
2016	53	11.2
2017	81	13
2018	126	14.8
2019	161	10.2
2020	81	10.2

*Source.* APAV (2016, 2017, 2018, 2019, 2020).

However, the same cannot be said for its criminalization. In Portugal, street harassment is considered a crime under Article 170° of the Portuguese Penal Code, through the criminalization of “exhibitionist acts,” “sexual touches,” and the “formulation of sexual proposals,” leading to 1-year incarceration or to a monetary fine up to 120 days. The Article exists since 2007 and was revised in 2015 following Portugal's rectification of the Istanbul Convention (Diário da República, 2015). However, despite the existence of this law, Portugal verifies a very weak criminalization of street harassment. Table 2 displays street harassment's arrests and investigations opened by this type of crime (percentage of the investigations opened for crimes against sexual freedom and self-determination), registered by the *Relatórios Anuais de Segurança Interna*.^5^ As shown, there have only been *seven* arrests for the crime of street harassment in the years 2016–2020.

**Table 2.** Street Harassment's Arrests and Opened Investigations in Portugal 2016–2020.

Year	Arrests (*N*)	Opened Investigations (%)
2016	2	5.4
2017	1	5.7
2018	4	6.1
2019	—	—
2020	0	3.8

*Source.* Sistema de Segurança Interna (2016, 2017, 2018, 2019, 2020).

This signalizes something is failing in street harassment's criminalization. Like many countries, Portugal verifies a very low rate of victim's reports of gender crimes. This is of particular relevance because street harassment constitutes a “semipublic crime” in national law. This means that contrary to “public crimes” (e.g., intimate partner violence), no investigation can take place without the victim has filed a formal complaint to the authorities (Procuradoria Geral Distrital do Porto, n.d.), which is almost always taken by the PSP. Its' workers thus have a preeminent role in the whole process, sustaining the need for an analysis of their actions.

### Street-Level Bureaucracy

According to Lipsky (1980), street-level bureaucrats are the state employees responsible for the daily implementation of public policies, whose work involves direct interaction with citizens. Some examples are health professionals, teachers, judges, and police officers (Lipsky, 1980; Maynard-Moody & Musheno, 2000; Tummers & Bekkers, 2012). These workers are faced with a constant dilemma between treating all citizens as equals, while, at the same time, remaining responsive to individual particularities (Lipsky, 1980; Tummers & Bekkers, 2012). This derives from street-level bureaucrats possessing high levels of *discretion*—that is, the freedom to decide what should be done in a given situation—and *autonomy*—that is, the ability to make your own decisions without being controlled by anyone else (Lipsky, 1980; Tummers & Bekkers, 2012).

Consequently, street-level bureaucrats can produce different outcomes at implementation than those expected (Kirby & Krone, 2002). Their actions can produce “street-level divergence”—that is, a gap between formal stated policy and street-level actions (Gofen, 2013). Three factors have been shown to cause this divergence. First, the content of a given policy (Higgs & Rowland, 2005; Tummers et al., 2012). Tummers et al. (2012) demonstrated a strong relationship between street-level bureaucrats' perception of a policy's content and the likelihood of them implementing it, arguing willingness to implement will increase the more (a) societal, (b) client, and (c) personal meaningfulness street-level bureaucrats attribute to a certain policy.

Second, following new institutionalism (Keiser et al., 2002), the institutional environment where the implementation occurs impacts individual action (Keiser et al., 2002; Tummers et al., 2012). Police institutions portray this phenomenon particularly well, as they are characterized by a prevailing masculinist culture (Brown & King, 1998; Prokos & Padavic, 2002; Silvestri, 2017) that highlights gender roles and stereotypes (Brown & King, 1998; Jamel, 2010; Lockwood & Prohaska, 2015). This often creates a misogynist environment, rooted in discrimination and sexual harassment (Brown, 2007; Collins, 2004) that has been shown to negatively affect the criminalization made by police officers of violence against women's crimes, particularly rape (McMillan, 2016; Rich & Seffrin, 2012; Spohn et al., 2015). Thus, the implementation of Article 170° at the level of the PSP's street-level bureaucrats can be affected by the institutional environment in which these workers are embedded.

Third, mobilizing the theory of representative bureaucracy (Kingsley, 1944), the personal characteristics of the street-level bureaucrats themselves (Keiser et al., 2002; Meier & Nicholson-Crotty, 2006; Riccucci & Saidel, 1997; Tummers et al., 2012; Wilkins & Keiser, 2004). In implementation, in order for passive representation—when an institution has the same demographic characteristics as the population they represent/serve (Mosher, 1982)—to evolve to active representation—when the bureaucrats of an institution actively defend the interests of the portion of the population that they passively represent (Mosher, 1982)—three conditions need to be fulfilled: (I) that the policy is implemented by bureaucrats possessing discretion and autonomy; (II) that the policy is prominent for a group that shares the same demographic characteristics; and (III) that its implementation benefits a certain group (Wilkins & Keiser, 2004). With this framework, women have been shown to actively defend women's interests and enhance implementation as teachers (Keiser et al., 2002), social workers (Wilkins & Keiser, 2004), and police workers (Meier & Nicholson-Crotty, 2006). How these three factors affect the implementation of street harassment's legislations is yet to be verified, and this investigation aims contributing to fulfill this gap.

## Method

A qualitative dataset was gathered through semi-structured interviews with officers from the PSP. A qualitative methodology was preferred because it allows seeing a social phenomenon—here, street harassment and its criminalization—through the eyes of the object of study (Bryman, 2012)—here, the PSP workers. Similarly, semi-structured interviews were chosen because they provide greater freedom of response and more space to develop points of view to interviewees than structured ones (Bryman, 2012). The questions were written based on the abovementioned literature and the script is provided in the Appendix.

A total of 14 interviews were conducted. The choice not to conduct more interviews was based on sample saturation (Hennink & Kaiser, 2022). The first interview was conducted in person and the other 13 over telephone^6^ from October 2020 to February 2021, lasting between 15 and 25 min. They were recorded with the authorization of the interviewees and transcribed ipsis verbis with anonymity secured. Of the 14 interviewees, 9 were men and 5 were women. No other sample characterization is presented (e.g., age or city) as requested by the officers themselves. Hence, both because I was specifically requested not to mention the cities they worked in, and because Article 170° is a national law, this is a national study. However, as these workers were selected through convenience and snowball sampling from a population of 20,337 officers (PSP, 2019), both non-probabilistic sampling techniques (Johnson et al., 2016), this is a non-representative study that does not generalize its conclusions to the entire population.

Efforts were made to reduce sample and investigation bias to ensure maximum axiological neutrality (Weber, 2004). Regarding sample bias, snowball mechanisms were only applied to those interviewees selected through convenience that was not within my network of friends (which were two of the three interviewees selected through convenience). This ensures the interviewees were not selected in order to confirm any inclination that I, even if unconsciously, could have. Regarding investigation bias, the interview questions were always neutrally formulated; furthermore, I only intervened to introduce a new question and always remained equally neutral in my posture during the whole interview.

A thematic content analysis was made to the dataset resulting from the interviews. For this, categories of analysis were created. These are: discretion/autonomy—emerging directly from Lipsky's theory (1980), aiming to confirm PSP workers as street-level bureaucrats and, therefore, as workers with agency; benefits of the law, with the subcategories social benefits, client benefits and personal benefits—emerging from the literature, as the perception of a law's content seems intertwined with the implementation process (Tummers et al., 2012); unawareness of the law—emerging inductively from the material, as it was generalized among the interviewees; masculine culture, with the subcategories adherence to myths and devaluation—emerging from the literature, as the widespread masculine culture of police institutions seems to impact its workers (Brown and King, 1998; Jamel, 2010; Lockwood & Prohaska, 2015; Moore, 2010; Prokos & Padavic, 2002; Silvestri, 2017); and prior experience of victimization—emerging from a hybrid between literature and the material, as the first indicates personal experiences impact implementation, and the interviewees themselves also alluded to this. Finally, although not as category, the gender of the interviewees was also included in the analysis.

A mixed analysis that combined quantitative and qualitative methods was developed. Regarding the first, the aforementioned analytical categories generated eight nominal variables that were organized in an Excel spreadsheet in terms of presence and absence in the 14 interviews. The gender of the interviewees was also added to that same sheet. Presence was only counted as such if the interviewee was referring to him/herself specifically, except in the category discretion/autonomy, where a reference to the work of other colleagues was also marked as present. Regarding the second, the analysis focused on the speech of the interviewees, their own points of view, and understanding of the object of study (Dantas, 2016). The text segments where the categories rose were isolated, allowing for a comparison and interpretation of the interviewees' discourses. For this purpose, an analysis grid was built, isolating the text extracts with reference to the categories created. Subsequently, conclusions were drawn inductively.

## Findings

Table 3 provides an overview of the analytical categories' presence in the interviewees' discourses. It is clear that not all categories have the same level of presence: *social benefits* of the law (100%) and *discretion/autonomy* (93%) registered the highest counts, followed by an *unawareness of the law* (79%) and an *adherence to myths* (64%). The subcategory *client benefits* (57%) and the category *prior experience of victimization* (50%) registered intermediate counts. Finally, the subcategories *personal benefits* (43%) and *devaluation* (29%) recorded the lowest frequencies.

**Table 3.** Presence of the Analytical Categories in the Interviewees' Discourses.

	Present		Absent	
Categories	*N*	%	*N*	%
Discretion/autonomy	13	93	1	7
Social benefits	14	100	0	0
Client benefits	8	57	6	43
Personal benefits	6	43	6	43
Unawareness of the law	11	79	3	21
Devaluation	4	29	10	71
Adherence to myths	9	64	5	36
Prior experience of victimization	7	50	7	50

### The PSP’s Agents as Street-Level Bureaucrats

The PSP's agents resorted to several references to the characteristics of discretion and autonomy when describing their daily work. Indeed, the category discretion/autonomy verified the prominent presence of 93%. For example, one interviewee stated that “the person at the counter receiving the complaint can immediately clarify. The person can move the means more quickly to aid the person” (E9)—suggesting discretion—whereas another stated that “the police work depends a lot on what you write. You can do anything depending on what you write” (E1)—suggesting autonomy. Overall, the interviewees clearly confirm Lipsky's (1980) argument that police officers are street-level bureaucrats.

As such, they maintain a constant balance between presenting the same attitude toward all complaints, while, at the same time, being sensitive to the particularities of each one. The interviewees alluded to this: “People are treated accordingly (…). It is one thing to complain about a flat tire, logically they are not mistreated, but if there is a person complaining about a sexual abuse, then the matter must be treated differently” (E8). Overall, it became evident that the PSP's workers possess agency during their work, being able to influence the implementation process and potentially rise obstacles to it.

### Obstacles to Implementation (I)—The Legislation

The officers also provided relevant data on their relationship with the legislation. As shown in Table 3, the category *social benefits* has a presence of 100%. This means that all interviewees attribute societal meaningfulness to the Article. According to them, the legislation rightfully signalizes that the behaviors that characterize street harassment must be penalized as a crime. However, not all interviewees attribute client or personal meaningfulness to the legislation. As portrayed in Table 3, only 57% of the interviewees believe the legislation is beneficial to its clients—that is, street harassment victims—and only 43% believe it to be beneficial to him/herself.

Regarding client meaningfulness, although some interviewees consider that the legislation automatically benefits the victims by existing (6/14), others do not consider this to be enough. More than half of the sample referred that the police does not have enough implementation instruments to effectively help the victims. Accordingly, some of the interviewees (2/14) referred to the difficulty they face in implementing this law due to victims not pressing charges: since street harassment is considered a semipublic crime, the police can’t investigate without a formal complaint from the victim. Others alluded to the difficulty they face in collecting evidence (7/14), particularly in identifying the suspects of the crime (5/14). In addition, there tends to be a lack of physical evidence (witnesses, images, videos, etc.), with the victim's word often being the only available evidence, often not enough to support a case (2/14). The accumulation of these difficulties led almost half of the interviewees to fail in attributing client meaningfulness to the law.

As for personal meaningfulness, eight of the interviewees do not regard the present legislation as beneficial to him/herself. Of these eight, three of them are men, and five of them are women (the total amount of women interviewed). Of the three men, two were unable to explain why they believe the legislation does not benefit them, and the other considers that, as the penalties applied are minor, the law ends up benefiting the perpetrators of street harassment; consequently, the law does not benefit him because he does not “intend to harass anyone” (E4).

Perhaps more interesting is the fact that all five women interviewed believe the law does not benefit them. This question was asked directly to all the interviewees, and all the women replied a clearcut “no.” The reasons for this were varied: some argued that filing a complaint for street harassment is inconsequent. For example, when asked if she had ever filed a complaint, one woman stated that “no, because (…) harassment of this type is in public space and comes from people you don’t know. So, who are we going to press charges against?” (E12); others recalled personal experiences with street harassment where nothing happened to their perpetrators—for example, “It has happened to me (…) and I didn’t feel that justice was done. I went to a disorder incident, and the individual was drunk, and said everything and anything of a sexual nature (…) But (…) that was not considered” (E11). Overall, all five women mentioned they prefer to deal with street harassment's occurrences alone rather than using the law: “if I benefit from the law for some reason it will be at a very high level, because I manage to solve it myself” (E10).

Finally, Table 3 shows that the category *unawareness of the law* has a presence of 79%. One of the interviewees admitted he was completely unaware of the content of Article 170°. Furthermore, two of the interviewees stated that street harassment is a public crime—and, therefore, that the police does not require a formal complaint from the victim; as aforementioned, this is a misconception. However, the biggest misunderstanding the interviewees demonstrated regarded what classifies as street harassment, with eight of them stating that *piropos*^7^ are formalized as crime under Article 170°, and one stating that it is limited to verbal acts only—“basically it is verbal … it does not imply physical or other aggressions. It is really just verbal things” (E11). As aforementioned, Portugal criminalizes “exhibitionist acts,” “sexual touches,” and “formulation of sexual proposals.” Of the three, only the latter accounts for street harassment's verbal expressions; furthermore, although *piropos* can take the form of a sexual proposal, the majority of them do not.

### Obstacles to Implementation (II)—The Institutional Environment

Parallelly, the interviewees also confirm the widespread finding that police institutions are characterized by a masculinist culture: the subcategory adherence to myths verified a presence of 64% (9/14), and the subcategory devaluation verified a presence of 29% (4/14). This indicates the presence of stereotyped notions about street harassment among the police officers interviewed. As a result, by aggregating the two subcategories in the main category male culture, the last verified a presence of 64%.

Regarding the first subcategory, it draws from the definition of “rape myths”—that is, “attitudes and generally false beliefs about rape that are widely and persistently held, and that serve to deny and justify male sexual aggression against women” (Lonsway & Fitzgerald, 1994, p. 133)—applying it to the particular case of street harassment. This means that 64% of interviewees adhered to false beliefs about street harassment that, even if unintentionally, ultimately result in a denial and/or justification of this type of harassment. Particularly present was the myth that women often lie about street harassment occurrences, especially in order to “get back” to a man/previous partner (5/14): “(…) those cases were nothing happened, and people are just trying to vilify the other person, making false statements and accusing an innocent person” (Interviewee 4):

But this leads to other situations, for example, a person who wants to harm someone … for example, a lady who wanted something [romantic] with a gentleman, and that gentleman did not want it. The women, resentfully, can make a video and set up a situation that looks like sexual harassment. (E5)

This idea that women take advantage of available legislation and often lie about street harassment mirrors one of the most widespread myths about rape: that women lie about having been raped, and make false statements about it (Rape Crisis, n.d.). Furthermost, one of the interviewees incurred in victim blaming in his discourse: “(…) people don’t always tell us the whole story, you know? (…) They can omit certain things. Maybe they did something that made them be the victim of sexual harassment” (E4). Again, blaming the victim, and believing that she “asked for it,” is a widely sustained myth about rape (Rape Crisis, n.d.).

Regarding the second subcategory, although less prevalent than the subcategory *adherence to myths*, at least some of the interviewees devaluated street harassment's severity. These interviewees considered that especially when compared to other crimes like sexual harassment in the workplace or rape, street harassment is not a severe crime. For example, one affirmed that “if a person experiences it in the workplace it is more serious (…) than having a sporadic and unknown stranger’s harassment” (Interviewee 4), while other stated that “[women] are not really bothered [with street harassment], as (…) he [the perpetrator] does not try to rape them” (E9).

**Figure 1.**
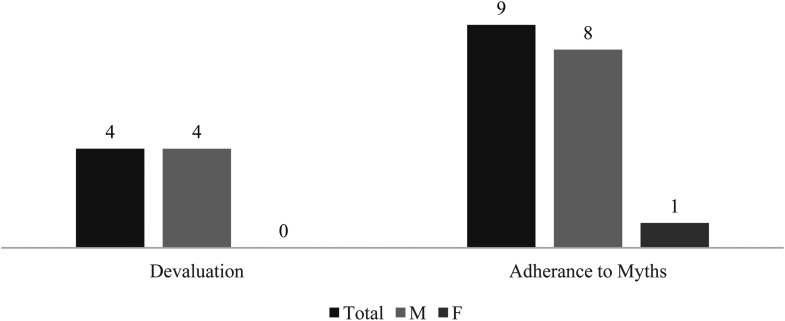
Presence of the Masculine Culture's Subcategories by Gender of the Interviewee.

Overall, the presence of the category masculinist culture verified a clear variation according to the interviewee's gender. As portrayed in Figure 1, it is the men that seem to contribute to the perpetuation of this culture in the institution: of the four interviewees who showed signs of devaluation, none of them are women, and of the nine interviewees who adhered to myths, only one corresponds to a female interviewee. Consequently, this masculine culture characterizing police institutions carries gender differences and, overall, was less visible among the women.

### Obstacles to Implementation (III)—The Workers

Literature shows how active representation is expected to happen during implementation when (a) the policy is implemented by bureaucrats with discretion and autonomy; (b) the policy is prominent for a group sharing a demographic characteristic; and (c) its' implementation benefits a particular group (Wilkins & Keiser, 2004). As aforementioned, Condition (I) is verified. As for Condition (II), the policy is prominent for the group “women,” with the shared characteristic being the “female gender.” Because it is a form of gender violence, street harassment disproportionally affects women, and the category *prior experience of victimization* confirms the already expected. As one of the women interviewed stated: “I am a women, I think all of us have suffered at least some kind of harassment” (E11). Indeed, the totality of the women interviewed (*N* = 5) affirmed having been victims of street harassment, compared to only two of the men interviewed (*N* = 9). As women are the main victims of street harassment, Article 170° is a prominent policy for them, and “female gender” is the shared demographic characteristic.

As for Condition (III), the conclusions are not so straightforward. Article 170°'s 2015 amendment followed Portugal's rectification of the Istanbul Convention. This Convention's main objectives are “a) To protect women against all forms of violence, as well as to prevent, to institute criminal proceedings in relation to violence against women and domestic violence and to eliminate these two types of violence” (Istanbul Convention, Article 1). In other words, it was *formulated* to benefit women. But this does necessarily translate to its *implementation* benefiting women.

**Figure 2.**
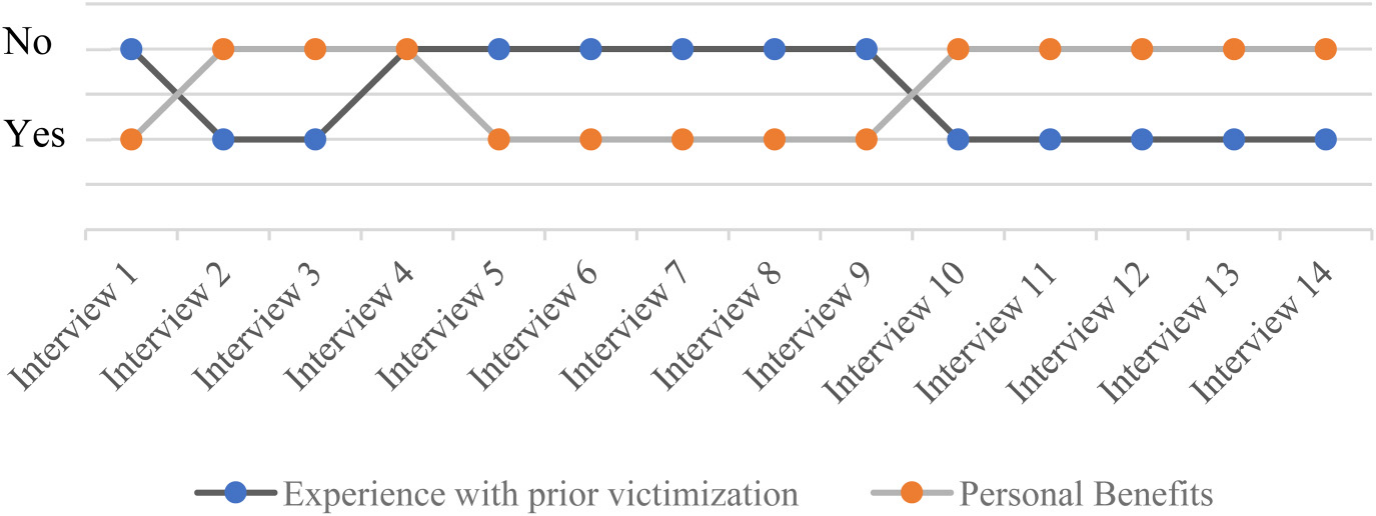
Correlation Between Prior Experience of Victimization and Personal Benefits.

Figure 2 correlates the subcategory *personal benefits* and the category *prior experience of victimization*. As shown, there is an almost perfect correlation between these two categories. Compared to only one of the interviewees who has not been the victim of street harassment and does not consider the law to be personally beneficial to him, all the interviewees who have been victims of street harassment do not attribute personal meaningfulness to it. Put differently: the men, who experience few situations of street harassment, are those who attribute more personal benefits to the law. On the contrary, the women, the common victims of street harassment, are those who don’t attribute personal benefits to the law. Furthermore, as aforementioned, there are clear gender differences among the subcategories *personal benefits* and *client benefits*. Although no generalizing conclusion can be drawn from such a small sample, at least according to the women interviewed, the present legislation does not benefit women as a group. Thus, notwithstanding the possibility of other positions, Condition (III) is not confirmed in the present study. To conclude, this means the present investigation did not find a clear case of active representation during implementation.

## Discussion

It is clear that the PSP police officers exercise their daily work with agency. According to the interviewees themselves, this causes differences in attitudes among workers accordingly to different situations. If, on the one hand, this can be regarded as desirable, for it allows an adjustable treatment to each case, on the other hand, it can compromise the implementation process. In fact, it was mentioned by the interviewees themselves that not all PSP workers treat victims of crimes the same way. Some stated that female workers are more sensitive in the treatment of victims of certain crimes, namely gender crimes, such as street harassment. They also referred that, because of this, female victims tend to feel more comfortable addressing their complaints to female workers:

The victims are women, as a rule they are more open with a female police element, because somehow they identify themselves and are not ashamed (…). They open up more. And sometimes they say things to us that maybe with an element of the male gender they wouldn’t say. (E12)

Overall, this poses a question of efficacy, as around the globe police forces are overwhelmingly masculine.

### Obstacles to Implementation (I)—The Legislation

If the content of a policy can work as an obstacle to its implementation, the perception that street-level bureaucrats have of it plays a fundamental role (Tummers et al., 2012). Specifically, the willingness to implement has been shown to increase the more meaningfulness street-level bureaucrats attribute to said policy, be it societal, client, or personal (Tummers et al., 2012). Thus, obstacles to the implementation of Article 170° can arise if PSP's workers do not regard it as meaningful. Indeed, as aforementioned, although all interviewees attribute societal meaningfulness to the legislation, not all of them attribute client or personal meaningfulness to it. As demonstrated in the literature (Tummers et al., 2012), this can affect the workers' willingness to implement the legislation, ultimately resulting in a flawed process.

**Figure 3.**
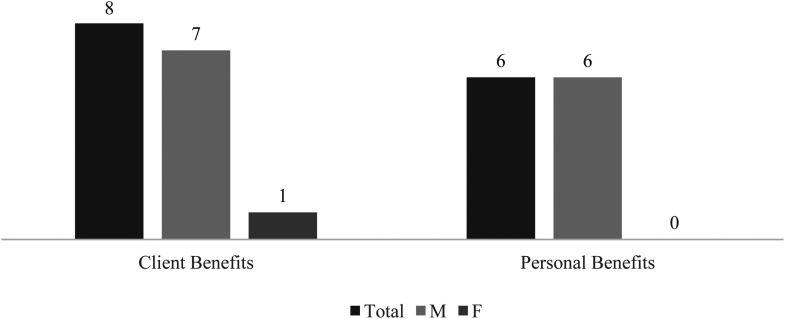
Presence of Subcategories Client Benefits and Personal Benefits by Gender of the Interviewee.

This became apparently evident among the female police officers. As shown in Figure 3, it is also possible to notice a three-cut relationship between gender, the perception of personal benefits, and the perception of client benefits. Not only none of the women attribute personal benefits to the law, but also only one attributes client benefits to it—that is, only one woman believes Article 170° to be beneficial to street harassment's victims. Having experienced the existing legislation as inconsequent to them as victims, these workers were skeptical of it benefiting other victims—“things don’t go as they should. There are a lot of delays, nothing goes forward, everything is archived (…). It doesn’t benefit women because it does absolutely nothing” (E13).

Parallelly, a large portion of the sample demonstrated some kind of faulty conception of the law. This too can pose an obstacle to its implementation: if PSP's street-level bureaucrats have a limited knowledge of Article 170°, its proper implementation can be compromised. The fact that a large number of interviewees showed signs of ignorance about the content of Article 170° also demonstrates a lack of training of the PSP's agents regarding the content of the laws they have to implement, which can lead to perceptions of the law that minimize what it actually penalizes or to a situation of total ignorance of the law. This poses an obstacle to the implementation process because one cannot rightfully implement something that one is not familiar with.

### Obstacles to Implementation (II)—The Institutional Environment

The masculinist culture characterizing police forces around the world also unraveled in the Portuguese PSP. This impacts the (re)construction of police officer's identities, often leading to an exaggeration of aggressive physical action, competitiveness, and a preoccupation with images of conflict and heterosexuality (Brown & King, 1998). One of the interviewees perfectly portrays this, having stated that “I do not intend to harass anyone … that is, except for my girlfriend [laughs]” (E4). Overall, attitudes such as these can create a misogynist work environment, where discrimination and sexual harassment thrive (Brown, 2007; Collins, 2004). Indeed, one of the female interviewees alluded to this, recalling having been a victim of sexual harassment in the workplace:

Sexual harassment exists, both outside and inside the institution. It is a world of men, and they always try. When we are placed new in a police station, of course everyone tries. It is like a drop of blood in an ocean full of sharks. And even a hierarchical superior, my commander-in-chief, actually harassed me once. I went to his office to solve a situation and he said «oh don’t worry, everything can be solved, I’ll take care of it». But then when I was leaving the office he said «but don’t forget, everything has a price. (E12)

One of this culture's main features is the widespread acceptance of myths about sexual violence. The way a great majority of the workers referred to street harassment occurrences and their victims mirrored the discursive framework of rape myths. This signals that the same myths that exist about rape can also exist about street harassment, raising the hypothesis that they may not be exclusive to the reality of rape, but perhaps to that of gender crimes. The presence of these myths among the PSP workers can hinder the implementation of Article 170° in two ways: when victims come forwards to file a complaint, and during the de facto investigation conducted by the police. When it comes to the first, adherence to myths about street harassment can tamper the receptiveness of police officers to victims about to file a complaint: these can be met with skepticism, disbelief, and/or insensitivity (Garza & Franklin, 2021). In fact, this possibility was recognized by the interviewees themselves, with one stating that

a lady goes to the police and is attended by a male police officer (…). There may be (…) a feeling of “I will go there, and I will say that I was harassed, but they might tell me that I was dressed a certain way….” There are some people who think like this, and it is normal, because a few years ago this would almost certainly happen. (E3)

Since in Portugal street harassment needs the victim's formal complaint, these kinds of attitudes from the PSP's workers could impact victim's willingness to file a complaint. There is an abundant body of literature showing this happens in other gender violence crimes, such as rape (Brown & King, 1998; Garza & Franklin, 2021; Jamel, 2010), but because the present study is not victim-centered, this can only be raised as a (very likely) hypothesis. Whereas for the second, these myths can also directly inhibit the investigation of a street harassment occurrence. If literature has already shown how police officers adhering to rape myths is linked to a smaller number of opening of cases, investigations, and arrests of suspects (Garza & Franklin, 2021), the same could also be happening with street harassment cases.

Similarly, the unawareness around the content of Article 170° extended to an overall unawareness of the violence behind street harassment. Not all of the PSP's street-level bureaucrats demonstrated being fully conscious of the consequences street harassment poses to women. As with adherence to myths, devaluation of street harassment's severity can also diminish the efforts to pursue its criminalization. This was in fact confirmed by two interviewees: “It is stated in the law. Now, if the law is applied or not, that is something different… Because sometimes people (…) devalue certain behaviours (…), as they do not feel it in their own skin” (E6):

it is not considered a serious crime. It is not considered important. (…) Basically, things will end there, there will be no investigation, nothing. You can’t reach the perpetrator, but you also don’t try like in a robbery. There are still descriptions in a robbery (…), follow-ups, spending more time on the streets … now when it comes to street harassment, if a person comes to tell an officer “I was harassed on that street, by person x, with characteristics y,” I don’t believe they would send us there to investigate the situation. (Interviewee 11)

### Obstacles to Implementation (III)—The Workers

Finally, according to the theory of representative bureaucracy, the gender of the implementors affects implementation and women have been shown to “act for women” during this stage (Keiser et al., 2002; Meier & Nicholson-Crotty, 2006; Wilkins & Keiser, 2004). However, the present study did not find this to be happening.

Contrary to previous findings, what is concluded is that both men and women can act as obstacles to implementation in this context; inversely, both can enhance implementation and “act for women.” On the one hand, the women interviewed demonstrated having their personal experiences with street harassment raise doubts about the efficiency of the law which, according to literature, increases the likelihood of non-implementation. On the other hand, women did not devaluate the severity of street harassment nor did they demonstrate adherence to myths about it, which can enhance implementation. At the same time, the men interviewed demonstrated higher levels of perception of client and personal benefits to the law—probably because they never had any real experience with it; this, according to the literature, increases the likelihood of implementation. However, it was the men who largely incurred in devaluation and adherence to myths about street harassment, which poses obstacles to implementation.

Contrary to what was found by Meier and Nicholson-Crotty (2006) regarding sexual assault in the United States, a greater number of women police officers may not mean a greater number of complaints and arrests for street harassment in Portugal. This contributes to the development of the theory of representative bureaucracy by raising suggesting active representation may be jeopardized due to systemic gender violence.

## Conclusion

This study unraveled several obstacles to the implementation of the legislation that criminalizes street harassment in Portugal among the PSP's workers. It was found that, although all implementors attribute societal meaningfulness to the legislation, not all consider it to have client or personal meaningfulness. This has been shown by previous studies to hinder implementation (Tummers & Bekkers, 2012). This also indicates a disparity between the recognition of practical and symbolic benefits of Article 170°, which tends to happen when the legislator addresses the symptoms of a certain problem—here, street harassment—instead of the root of its cause (McConnell, 2010)—the reproduction of a patriarchal society through gender violence.

Furthermore, a great majority of the officers interviewed demonstrated a lack of knowledge regarding Article 170°'s content and applicability. This resonates with Ventura's (2018) understanding of Portuguese society considering street harassment as a “not so serious offense.” This is also evident in the widespread adherence to myths and devaluation demonstrated by the interviewees’ discourses. The Portuguese PSP appears characterized by a masculine culture also found in other police forces of the world (Brown & King, 1998; Jamel, 2010; Lockwood & Prohaska, 2015; Moore, 2010; Prokos & Padavic, 2002; Silvestri, 2017), that has been proven to hinder implementation (McMillan, 2016; Rich & Seffrin, 2012; Spohn et al., 2015). It also creates a misogynist work environment rooted in discrimination and sexual harassment (Brown, 2007; Collins, 2004), as demonstrated by the women interviewed.

Finally, this study found that both men and women workers can hinder implementation, not confirming the theory of representative bureaucracy. Contrary to what was expected according to the literature (Meier & Nicholson-Crotty, 2006), a greater number of women police workers may not necessarily mean a greater number of complaints and arrests for street harassment. Despite having confirmed a gender disparity in the (de)valuation of this crime, with male workers being associated with greater devaluation, this did not translate into a greater willingness to implement among female workers. It was found that the personal experience female police officers have with street harassment creates disbelief about the efficiency of the available legislation, jeopardizing implementation. This contributes to the theory of representative bureaucracy by suggesting gender violence's scars may impact women's active representation.

In the end, the implementation of the legislation that criminalizes street harassment in Portugal proved anything but obvious. The PSP's street-level bureaucrats seem not only to *face*, but also to *create* obstacles to the implementation of Article 170°. It also became visible for the first time in Portugal that the few complaints that may reach the police can be received with a frivolous and/or negligent attitude. This confirms the hypothesis that police workers are not particularly sensitive to gender crimes complaints, and that this generates a lack of trust among citizens—particularly women. These are new findings that not only contribute to an understanding of the perpetuation of gender violence in society as a whole, but also in state's institutions in particular.

Future investigations can build on this study to further develop its ideas, either by expanding it to a representative sample of the PSP or by including street-level bureaucrats of other institutions participating in the criminalization of street harassment. Parallelly, this framework can be applied to other countries. If obstacles to implementation among street-level bureaucrats are found across different settings, this could help explain the generalized inefficiency of street harassment's criminalization.

Although inefficient, the criminalization of street harassment is usually considered a positive step in the long journey to achieve gender equality. By criminalizing acts of “sexual harassment perpetrated in the public sphere,” the legislator signals this type of behavior is not acceptable. However, these laws will never eradicate its perpetuation alone. Because it is a gender crime, street harassment's existence derives from the unequal distribution of power between men and women in our society, which does not cease to manifest itself during implementation. It is therefore essential that political powers invest in holistic assessments of existing laws, incorporate obstacles found during implementation, and invest in a gender comprehensive training among its institutions’ workers. Women's security and full citizenship depend on so.

## Appendix

Interview's Script

1. Gender
2. Can you tell me a little bit about how your work goes on a daily basis? What tasks do you have, for example?
3. How would you define street harassment?
4. What do you think about the criminalization of street harassment in Portugal?
5. What is your experience with the law on street harassment?
  - Have you ever received a complaint for this crime? If so, how many?
  - And what about your colleagues?
  - When you receive a complaint for this crime, what do you usually do? Can you give me a practical example?
  - Do you think people who make a complaint are generally well received?
  - Do you think that everyone who makes a complaint is treated equally? If not, why?
  - Do you think the person receiving the complaint can make a difference in this treatment?
  - Do you see any patterns in people who are the target of street harassment? What about people who make formal complaints about it?
  - Do you think the law is effective? Why?
6. What do you think about the existence of this law?
  - Do you think it is a positive thing that street harassment is considered a crime? Why?
  - If so, do you think it is positive that it is so through this specific law? Why?
  - Do you think this law benefits people who are targets of street harassment?
  - Do you think this law benefits you?
  - If you could change something about this law, what would it be?

## Notes

1. Street harassment is only considered a crime in Australia, Austria, Belgium, Bulgaria, Croatia, Czech Republic, Denmark, Estonia, Finland, France, Greece, India, Irland, Israel, Malta, Mexico, Nepal, Poland, Portugal, Slovakia, New Zeeland, South Africa, the United Kingdom, and Turkey (c.f., EIGE, n.d.).

2. The widespread fear women have of being victims of sexual assault, especially of rape, casts its shadow over a generalizing fear of all types of crimes (Junger, 1987; Macmillan et al., 2000; Mellgren & Ivert, 2019).

3. APAV is a nonprofit organization that aims to contribute to the information, protection, and support of citizens who are victims of criminal offenses. Every year it produces a report showing the numbers of received complaints by type of crime team, by type of victim, and by type of offender.

4. With the exception of 2019, year to which there are no available data for street harassment.

5. Annual reports that present the values of reported crimes by various entities that make up the Portuguese Homeland Security System.

6. The decision to switch the interviews to the telephone was made due to a worsening of the COVID-19 pandemic, which made it necessary to restrict face-to-face social contacts.

7. Expression or phrase addressed to someone, usually to demonstrate physical appreciation, is known in English as *catcalling*.
